# Image Clarity Affects Tip-of-the-Tongue Rates for Faces

**DOI:** 10.3390/jintelligence11070135

**Published:** 2023-07-07

**Authors:** Hyeonjeong Lee, Ali Pournaghdali, Bennett L. Schwartz

**Affiliations:** 1 Department of Psychology, Florida International University, Miami, FL 33199, USA; hlee053@fiu.edu; 2 Leonard Davis School of Gerontology, University of Southern California, Los Angeles, CA 90007, USA; pournagh@usc.edu

**Keywords:** tip-of-the-tongue states, metamemory, metacognition, fluency, face memory

## Abstract

Tip-of-the-tongue states are subjective experiences that unrecalled target words will be remembered. This study investigates if the visual fluency of familiar faces affects the likelihood of tip-of-the-tongue experiences (TOTs) as well as name recall and name recognition. To manipulate visual fluency, three levels of clarity for 396 celebrity faces were set: high, medium, and low clarity. Four hundred and twenty-nine participants were asked to recall the last names of the celebrities for all clarity levels, and, if they did not recall, to indicate if they experienced a TOT. Following the TOT question, they performed a name recognition test. Results showed that higher-clarity faces resulted in higher TOT rates than lower-clarity faces for unrecalled faces. Name recall was also higher for clearer faces. However, clarity level did not affect the correct answer rate on the name recognition test. These results support the view that perceptual cue-based factors influence TOT experiences.

## 1. Introduction

A common experience is that we sometimes struggle to name a face that we recognize. That is, we may not be able to recall the name even though we feel that we know it (Brédart 2017; Yarmey 1973). The tip-of-the-tongue (henceforth, TOT) phenomenon refers to the temporary state in which we feel that we know a word or name, but we cannot access the information that we want to recall in the moment (Brown 1991; Brown and McNeill 1966). Like other metacognitive judgments, such as feeling-of-knowing judgments or judgments of learning, a TOT is a subjective experience. By subjective experience, we mean that the person has a strong feeling, in this case that the item is known, regardless of what is actually recalled or recognized. Diary studies show that TOTs are common for the names of people, both those close to the person and those who are famous to the public (Schwartz 2002). In addition, research on TOTs shows that they occur across a wide range of stimuli and circumstances and are conserved across language and culture (Schwartz and Metcalfe 2011) and occur both individually and in groups (Rousseau and Kashur 2021).

Current theory proposes that TOTs are based on heuristic processes (Cleary 2019; Huebert et al. 2022; Schwartz and Pournaghdali 2021). Heuristics are cognitive processes utilized to reach decisions by means of mental shortcuts. The research finds that TOTs are caused by processes other than the processes that direct retrieval (but see Gollan and Brown 2006). That is, TOTs reflects the processing of accessible information to assess what is likely represented, but not accessible, in memory. These processes include the familiarity of the cue, the retrieval of semantically related information, and the retrieved emotional information (Cleary 2019; Schwartz and Pournaghdali 2021).

In the past, most researchers have assumed that TOTs are a function of knowledge about a missing target (see Brown 2012, for a review). However, even the evidence that suggested a role for target knowledge in the formation of TOTs is now being questioned (Huebert et al. 2022). For example, one of the most widely held beliefs about TOTs is that they are influenced by the retrieval of partial information (Brown 2012). That is, people retrieve phonological or syntactic aspects of a missing word, and this retrieval information drives the TOT. However, Huebert et al. showed that the accuracy of retrieved information does not vary from TOTs to n-TOTs. Moreover, they showed that when people are in TOTs, although they are more likely to report such information, it is not more accurate. And when participants are forced to report information during TOTs, much of this information tends to be inaccurate. Thus, they challenge one of the most accepted versions of TOT causation.

Similarly, Cleary and Claxton (2015) also showed that an earlier presentation of the to-be-recalled target of a cue did not influence later TOT rates, regardless of the font size or visual salience of the target word. They studied the relation between fluent processing of the target’s physical features and TOTs by asking participants to make inferences about earlier answers to general knowledge questions. Cleary and Claxton showed that when people experience TOTs, participants judge that previously seen stimuli were visually easy to process. In their study, participants studied 40 answers out of 80 general knowledge questions on a white background screen: 20 answers with a dark black font as a high-clarity condition and those with a light gray font as a low-clarity condition. Similarly, in another experiment, they set large and small font size conditions in the same way as the font color. Then, in both experiments, participants were asked to answer the general questions and to report whether they experienced TOT states or not when they tried to answer the questions. Then, they rated how much clearer the font of the answer was regardless of whether they had studied the answer or if they had correctly answered the question. Cleary and Claxton’s results showed that participants responded that the font was clearer and bigger when they experienced TOTs in unrecalled answers than when they did not. This finding shows that being in a TOT influences how people process other related experiences. However, the actual font size of the targets did not influence TOT rates later when only the cue was presented. Thus, mounting evidence shows that neither access to an unretrieved target nor retrieved target information actually elicit TOTs (but see Schwartz and Smith 1997). For this reason, a further look at the effect of cues on TOTs is called for.

The current study focused on the role of cue fluency in influencing TOT experiences. Cue fluency means the ease with which the presented faces are processed by participants. More specifically, the higher the clarity of the face, the more fluently it will be processed. Our study aimed to determine if changes in TOT rates could occur solely by manipulating the visual fluency of cues. Making cues more familiar in cue priming studies has already been shown to influence TOTs for both word pairs and line drawings of animals (Metcalfe et al. 1993; Schwartz and Smith 1997). However, there have been no new empirical data on the effect of cues on TOTs in over 25 years, and the effect of cues on TOTs has not been looked at for face stimuli. Thus, because of the continued failure to find target effects (e.g., Huebert et al. 2022) and the lack of new studies on cue effects in TOTs, this study represents a reboot of an important area. To reiterate, examining the effect of cue fluency on TOTs for famous faces has not been studied previously.

We used celebrity faces as stimuli to induce TOTs. There are two advantages to using celebrity faces. One is that participants should have some familiarity with celebrity faces, and secondly, using celebrity faces provides ecological realism because people are expected to retrieve names in response to faces all the time. Celebrity faces are the faces that people do not see often but that most people will have some knowledge of. Thus, celebrity faces provide an excellent means to look at the relation of visual clarity and TOTs. Therefore, the current study examines how one’s visual fluency of a face might influence TOTs, name recall, and name recognition by using the faces of well-known celebrities.

In the current research, face–name stimuli were used to examine the effects of fluency on TOTs. Familiar faces as a stimulus have the following additional methodological merits: first, famous faces do not require participants to memorize faces and face-related information, so it is not necessary to consider encoding performance issues. Second, in addition to semantic memories of the face–name association, people may have related information already represented, such as that the celebrity appeared in a certain movie or that the person is an athlete. In other words, when recognizing a familiar face, people naturally retrieve knowledge about that person (Gobbini and Haxby 2007). Third, familiar faces may not be stored only as individual components. According to Piepers and Robbins (2012), a face is likely to be processed in a high-dimensional manner by considering not only the individual features of the face but also the interrelationships of those features as a whole. Østergaard Knudsen et al. (2021) assert that there is consensus among researchers that faces are processed holistically, at least when compared to other objects. Thus, visual fluency may affect the holistic perception of the face, thus leading to differences in memory performance.

There are several studies that have used famous faces as stimuli for eliciting TOTs. Several are event-related potentials studies of the relation between celebrity face naming and TOTs (Buján et al. 2012; Díaz et al. 2007; Galdo-Alvarez et al. 2009; Lindín and Díaz 2010), focusing on whether TOTs and retrieval show similar brain-wave patterns. Similarly, Kurosaki et al. (2022) showed that skin conductance responses were related to TOTs for famous faces. Other studies focused on the effect of cues on celebrity name recall (Hanley and Cowell 1988), the recall failure of face–names (Brédart and Geurten 2020), and the effect of facial movements on name recognition (Lander et al. 1999). None of these studies, however, manipulate the perceived fluency of the face being used as a stimulus for name recall.

The current study examined how fluent visual information processing of familiar faces affects TOTs for the names of those faces. The primary hypothesis, based on previous TOT studies, was that a visually clear famous face will elicit more TOTs of unrecalled names than less clear faces because visually clear faces will be processed fluently. With respect to recall, there are two potential outcomes. Our original hypothesis is that the clarity of a face will not affect the ability to access the name because faces are processed holistically and there should be sufficient clarity in even our low-fluency stimuli to allow this access to happen. However, if the greater clarity provides people with more information about the person, then name recall will be higher for clearer faces. That is, if greater clarity yields more related information, some of that information might trigger the name of the face. The same two possibilities exist for the recognition of the name following unsuccessful recall of the name. Higher clarity may or may not result in better name recognition. To be specific, when name options are presented in a name recognition test, the integration of visual fluency with name options that are directly related to target information may serve as a strong cue for retrieval. On the other hand, it is also possible that name recognition may not be influenced by visual fluency if the suggested name options serve as more useful retrieval cues than visual fluency. In summary, the main hypothesis of this study is that there will be more TOTs for more clear faces.

## 2. Materials and Methods

### 2.1. Participants

A total of 429 undergraduate students (55 males, 367 females, 7 others, M_age_ = 21.5 years, SD = 5.05) from Florida International University, Barnard College, and Columbia University participated in this study. There were 169 Barnard College and Columbia University and 260 Florida International University students in this study, and there were no significant differences among the three groups of undergraduates in the rate of recall, the number of TOTs, and recognition performance. Therefore, we do not report the data separately for each student group. Sixteen participants were excluded from the analysis because they did not complete the experiment, or they completed the experiment two or more times. Participants participated in the experiment through the SONA system (an online platform for participant recruitment) of their own schools and received credits corresponding to each school standard (one credit per hour in Florida International University and two credits per hour in Barnard College and Columbia University) after completion of the experiment. The maximum credits they could receive were two and four credits, respectively.

As this study was run completely online, participants were able to access the experiment from their own computers. Participants used their own laptops or desktop computers to participate in the experiment, but the use of smartphones was not allowed. Three hundred and fifty-five participants participated in the experiment using a laptop, sixty-two participants using a desktop, and seven participants using a tablet PC.

### 2.2. Stimuli and Procedures

**Figure 1:**
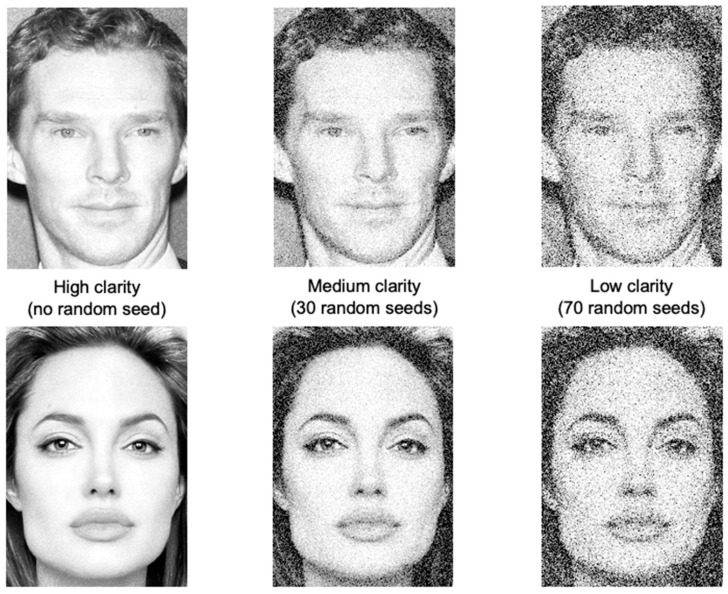
Examples of celebrity face photos and stimulus distribution per condition.

The stimuli used in this study were 200 × 300 pixel black-and-white frontal-face photos of 396 celebrities (198 women) taken from the “Celebrity Face Recognition Dataset” (https://github.com/prateekmehta59/Celebrity-Face-Recognition-Dataset, accessed on 2 January 2022. Each face photograph was presented at three different levels of clarity: high, medium, and low (see Figure 1). GIMP 2.10 was used to adjust the clarity of the images. This allowed us to blur faces in a consistent and constant way (see Figure 1). We manipulated the clarity of each face by adding a random noise filter to each image using the GIMP software. To this end, we added an RGB filter with the random seed value of 30 to the pictures in the medium-clarity condition and an RGB filter with the random seed value of 70 to the pictures in the low-clarity condition. Participants saw the 396 photos in a random order with the clarity of each photograph also randomized within each session. Therefore, each participant saw one and only one version of each of the 396 celebrities, and the randomization resulted in exactly one viewing by a participant of one of the three distinct versions of the faces. The experiment was conducted on the Qualtrics platform.

The experiment consisted of two separate sessions. Participants saw 198 celebrity faces in each session. The 198 photos included 66 high-clarity faces, 66 medium-clarity faces, and 66 low-clarity faces. The two sessions were assigned to each participant in a random order. We conducted two sessions to reduce fatigue for the participants.

**Figure 2:**
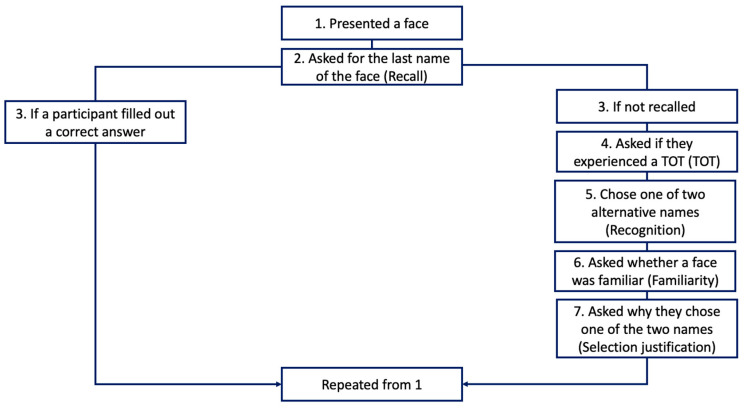
Schematic of the experiment session.

For each face, participants were asked whether they knew the last name for that face. We chose last names only to limit the amount of text each participant had to type in and to have the task be easier than it would have been if participants were required to type both first and last names. All famous faces were people who use both first and last names. When they typed the correct answer in the name recall test, they moved on to the next stimulus without further questions (Figure 2). The program could only catch some misspellings, and therefore, for some stimuli, the participant knew the answer but was asked about TOT and recognition anyway because their spelling was incorrect. Spelling was checked later by the first author and when the spelling was ambiguous, a second coder was consulted. If a misspelling was close enough to the correct answer, then it was counted as correct recall and subsequent responses were removed.

If they could not recall the name, they were asked if they were experiencing a TOT for that face. The exact instructions were “The tip-of-the-tongue (TOT) state is the feeling that you know the name and feel like you will recall it soon. Are you experiencing a TOT for this photo?”. TOTs were collected without the participant seeing the face but immediately after the recall test. Participants responded by typing in a “y” for a TOT or “n” if they were not in a TOT. Immediately following the TOT judgment, participants again saw the face, and they completed the recognition task. Recognition was assessed with a 2-choice discrimination task consisting of the name of the target and another name chosen to be similar to the target. Participants were asked “What is the last name of this person.” For example, one target name and one alternative name were provided considering ethnicity such as Lee and Liu when Bruce Lee was presented. Following the recognition task, participants were asked whether they felt that the person was familiar (yes or no), and then how they came to their recognition choice, in what we called the selection justification task. Participants were given four potential reasons for how they had made their decision in the recognition tests. The list of potential reasons is as follows: 1. “Now I remember the name”, 2. “I guessed the name and I don’t know who the person is”, 3. “I guessed the name, but I do know who the person is”, and 4. “I’m sure the other name option is not correct”. Following the selection justification task, the participant moved on to the next face.

## 3. Results

As each participant had seen one and only one version of each face and there was one experimental variable, the clarity conditions (three levels of clarity—high, medium, and low), a one-way repeated measures ANOVA was conducted to determine if the level of clarity affected the rate of name recall, TOTs, and face name recognition performance. We tested a large number of participants, and as such, we were concerned that some of our effects might be very small and a function of sheer size. Therefore, we randomly selected a set of 50 participants from the 429 participants. We then ran all the analyses on each subset. In each of these three analyses, the recall, TOT, and face name recognition data matched the full set. Thus, the results reported in this paper are based on the data from all 429 participants. Bayesian repeated measures ANOVA was performed as well, so the Bayes factors will be also reported with *p*-values. 

### 3.1. TOTs

**Figure 3:**
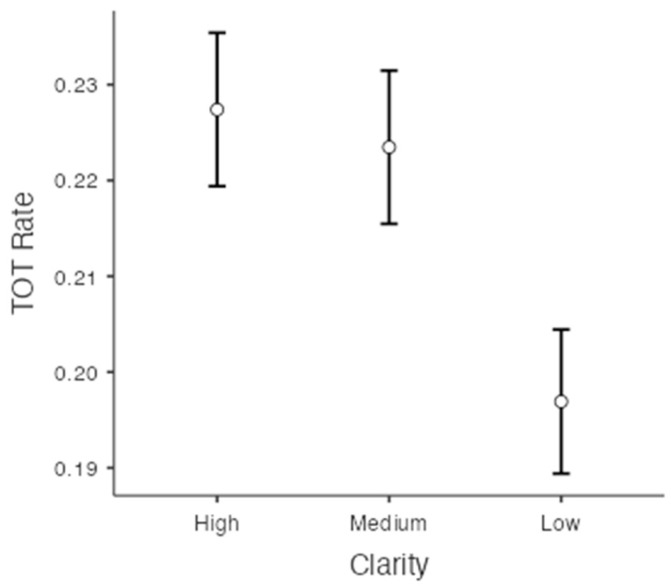
TOT rates as a function of on the levels of clarity.

TOT rate was defined as the number of TOTs divided by the number of all unrecalled items (see Schwartz and Pournaghdali 2021, for a justification of this procedure, and Gollan and Brown 2006, for extended discussion). The TOT rates for each clarity were 22.7% (SE = 0.008) in the high-clarity condition, 22.3% (SE = 0.008) in the medium-clarity condition, and 19.7% (SE = 0.008) in the low-clarity condition. There was a significant difference in the frequency of TOTs depending on the level of clarity, F(2, 856) = 74.451, *p* < .001, $\eta_{p}^{2}\;$= 0.15, *BF*_10_ = $1.76\times 10^{27}$. Bonferroni post hoc comparisons and Bayesian post hoc comparisons showed that the TOT rate in the high-clarity condition was significantly greater than the rate in the low-clarity condition, t(428) = 10.80, *p* < .001, *d* = 0.19, *BF*_10_ = $9.60\times 10^{20}$. The TOT rate in the medium-clarity condition was also significantly greater than the rate in the low-clarity condition, t(428) = 10.11, *p* < .001, *d* = 0.16, *BF*_10_ = $3.41\times 10^{18}$. However, there was no significant difference between the TOT rate in high- and medium-clarity conditions, t(428) = 1.46, *p* = .44, *BF*_10_ = 0.16 (see Figure 3). That is, participants reported fewer TOTs in the blurriest condition (low clarity) compared to the other two clarity conditions.

### 3.2. Recall

Recall was only considered correct if the participant recalled the correct last name. As indicated in the Materials and Methods, when a name was misspelled, we considered it correct, if coders agreed that it should be marked correct.

**Figure 4:**
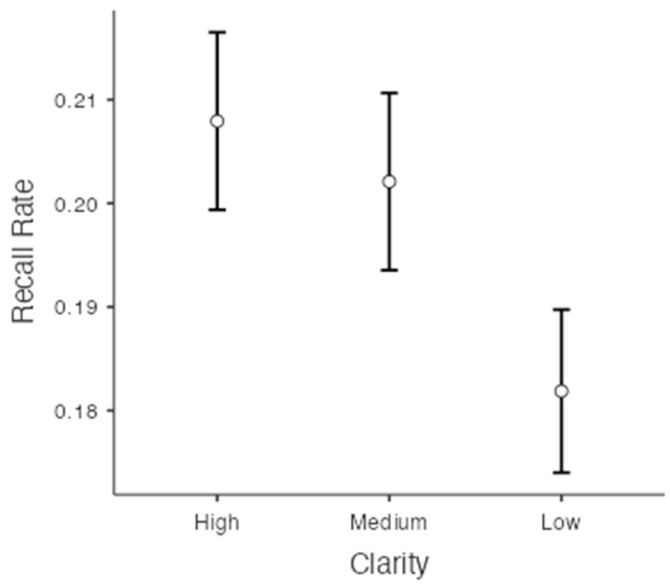
Recall rates as a function of on the levels of clarity.

Greenhouse–Geisser adjudgments were used in these analyses because the sphericity assumption was not met (*p* = .001). The recall rates for each clarity were 20.8% (SE = 0.009) in the high-clarity condition, 20.2% (SE = 0.009) in the medium-clarity condition, and 18.2% (SE = 0.008) in the low-clarity condition. We found a significant difference in the recall rate depending on the levels of clarity, F(1.93, 827.27) = 75.87, *p* < .001, $\eta_{p}^{2}\;$= 0.15, *BF*_10_ = $5.92\times 10^{27}$ (see Figure 4). Bonferroni post hoc comparisons and Bayesian post hoc comparisons showed that the recall rate in the high-clarity condition was significantly higher than the recall rate in the medium-clarity condition, t(428) = 2.90, *p* < .01, *d* = 0.03, *BF*_10_ = 3.41, and the recall rate in the low-clarity condition, t(428) = 11.02, *p* < .001 *d* = 0.15, *BF*_10_ = $6.25\times 10^{21}$. The recall rate in the medium-clarity condition was also significantly greater than the rate in the low-clarity condition, t(428) = 8.91, *p* < .001 *d* = 0.12, *BF*_10_ = $2.92\times 10^{14}$.

### 3.3. Face Name Recognition

The following analysis examined whether clarity affected face name recognition. Given that TOT judgments preceded the recognition test, we were able to investigate if both the clarity and the presence or absence of a TOT effected recognition accuracy. A two-way repeated measures ANOVA was conducted with post hoc comparisons by obtaining the recognition rate as a function of clarity and TOT, as was Bayesian repeated measures ANOVA. The presence or absence of a TOT is considered a quasi-experimental variable for the analysis. Specifically, a 3 × 2 within-subject ANOVA was conducted to examine the impact of clarity (high, medium, and low) and TOT experience (TOT and n-TOT) on the recognition rates.

**Figure 5:**
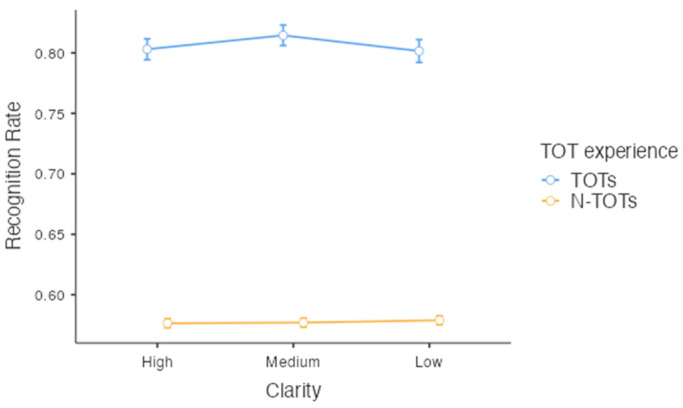
Recognition rates as a function of on the levels of clarity.

Greenhouse–Geisser adjustments were used in the analysis on recognition rates because the sphericity assumption was not met (*p* = .01). For stimuli for which participants experienced TOTs, the recognition rates for each level of clarity were 80.3% (SE = 0.009) in the high-clarity condition, 81.5% (SE = 0.009) in the medium-clarity condition, and 80.2% (SE = 0.009) in the low-clarity condition. For stimuli for which participants did not experience TOTs, the recognition rate for each level of clarity is 57.6% (SE = 0.004) in the high-clarity condition, 57.7% (SE = 0.004) in the medium-clarity condition, and 57.9% (SE = 0.004) in the low-clarity condition. There was a significant main effect for TOT experience (F(1, 428) = 1234.74, *p* < .001,$\;\eta_{p}^{2}$ = 0.743, *BF*_10_ = $2.69\times 10^{270}$) but not clarity (F(2, 838.96) = 0.95, *p* = .39, *BF*_10_ = $1.76\times 10^{-126}$). That is, participants performed better in the recognition test when they experienced TOTs (see Figure 5). There was no significant interaction, *p* = .29, *BF*_10_ = $4.27\times 10^{-4}$.^1^

### 3.4. Additional Measures

**Figure 6:**
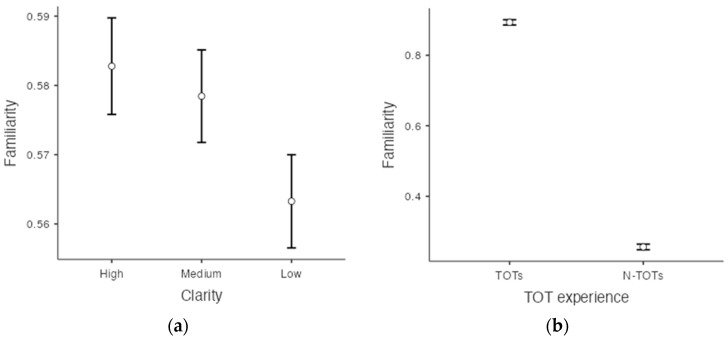
(**a**) Familiarity ratings as a function of the levels of clarity. (**b**) Familiarity ratings as a function of presence of a TOT.

The same analysis method used to assess recognition was used again to investigate whether both clarity and TOT experience affected the judgment of familiarity with famous faces. Familiarity was examined to determine if there was a correlation between perceived familiarity at the time of recognition and TOTs following recall. The familiarity analyses also used Greenhouse–Geisser adjustments as these analyses did not satisfy the sphericity assumption (*p* < .001). There were both significant main effects for TOT experience (F(1, 428) = 5161.09, *p* < .001, $\eta_{p}^{2}$ = 0.923, *BF*_10_ = 0.011) and clarity (F(1.91, 817.15) = 10.79, *p* < .001, $\eta_{p}^{2}$ = 0.025, *BF*_10_ = $7.16\times 10^{-238}$). TOTs were more likely to precede high-familiarity ratings. Bonferroni post hoc comparisons and Bayesian post hoc comparisons showed that the familiarity rate in the high-clarity condition was significantly different from the rate in the low-clarity condition, t(428) = 4.15, *p* < .001, *d* = 0.11 *BF*_10_ = 169.44, and the familiarity rate in the medium-clarity condition was also significantly different from the rate in the low-clarity condition, t(428) = 3.88, *p* < .01, *d* = 0.09, *BF*_10_ = 51.42 (Figure 6a,b). However, there was no significant interaction, *p* = .59, *BF*_10_ = 0.018.

**Figure 7:**
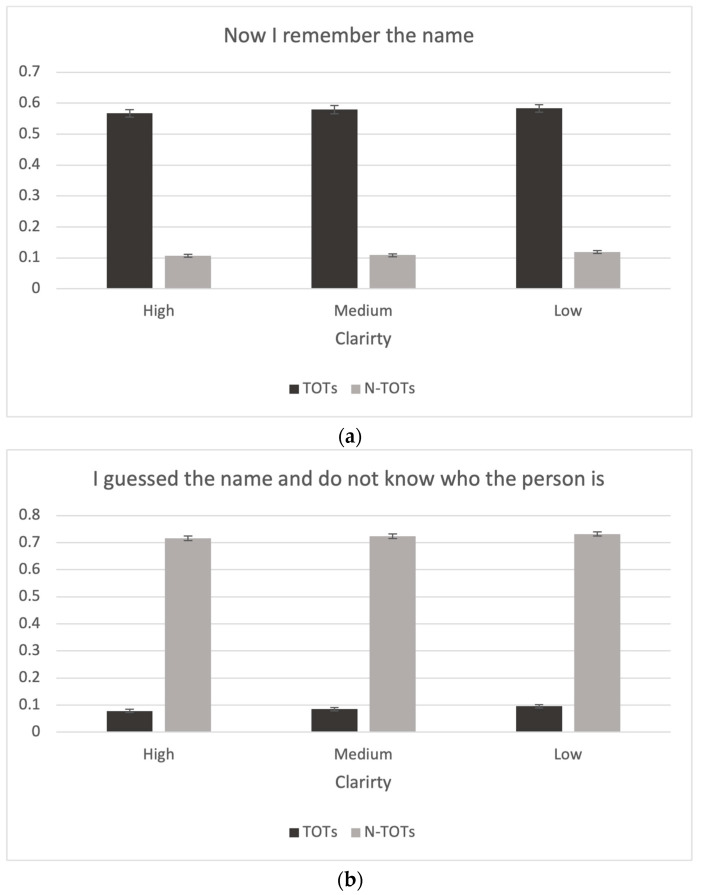

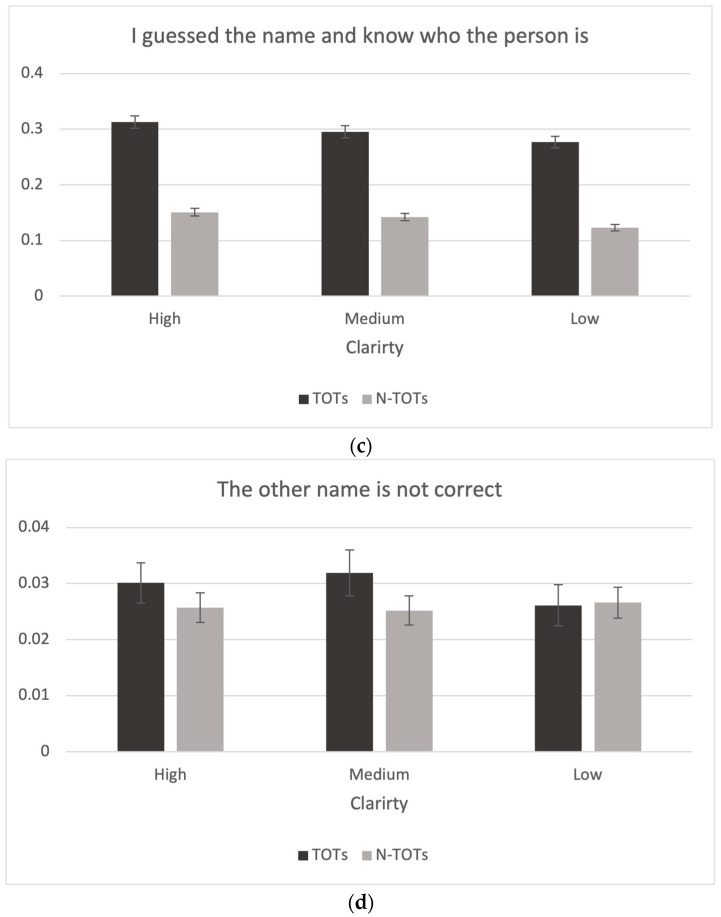
Choice in selection justification task as a function of clarity. (**a**) “Now I remember the name” judgments as a function of clarity condition and presence of a TOT. (**b**) “I guessed the name and do not know who the person is” judgments as a function of clarity condition and presence of a TOT. (**c**) “I guessed the name and know who the person is” judgments as a function of clarity condition and presence of a TOT. (**d**) “The other name is not correct” judgments as a function of clarity condition and presence of a TOT.

Lastly, the descriptive statistics and the bar graphs analyzing the selection justification task with four reasons for choosing a specific name in the recognition test are presented in Figure 7. Participants were likely to choose the reason “Now I remember the name” when they experienced a TOT more often than when they did not have a TOT, t(428) = 48.5, *p* < .001, *d* = 2.36, *BF*_10_ = $4.91\times 10^{175}$. Interestingly, participants chose “Now I remember the name” more often in the low-clarity condition than in the high-clarity condition, t(428) = −2.55, *p* < .05, *d* = −0.07, *BF*_10_ = 0.96 (see Figure 7). In contrast, participants chose the reason “I guessed the name and I don’t know who the person is” more often when they did not experience a TOT than when they did, t(428) = −74.5, *p* < .001, *d* = −4.06, *BF*_10_ = $9.38\times 10^{243}$. They also chose that reason more often in the low-clarity condition than in the high-clarity condition, t(428) = −4.58, *p* < .001, *d* = −0.11, *BF*_10_ = 785.44, as well as in the medium-clarity condition, t(428) = −2.83, *p* < .05, *d* = −0.06, *BF*_10_ = 1.95. The reason “I guessed the name but I do know who the person is” showed the opposite pattern to the reason “I guessed the name and I don’t know who the person is.” Participants chose the reason more when they experienced a TOT than when they did not, t(428) = 13.2, *p* < .001, *d* = 0.85, *BF*_10_ = $1.41\times 10^{14}$. They also chose this reason more often in the high-clarity condition than in the medium- and low-clarity conditions, t(428) = 2.78, *p* < .05, *d* = 0.07, *BF*_10_ = 1.51 and t(428) = 6.81, *p* < .001, *d* = 0.18, *BF*_10_ = $6.36\times 10^{8}$, respectively, as well as when comparing the medium-clarity condition to the low-clarity condition, t(428) = 4.14, *p* < .001, *d* = 0.10, *BF*_10_ = 178.01. Participants chose the reason “I’m sure the other name option is not correct” in only 2.8% of the trials, but there was no significant difference with respect to either TOTs or the clarity variable. We also found that in some trials people still “guessed” when they saw the name options after experiencing a TOT during recall. We had anticipated that participants would choose the correct name without hesitation after seeing the name when they experienced a TOT. However, it may be that, for some items, participants may have been in TOTs for names other than the actual one.

## 4. Discussion

This study investigated the effect of visual fluency of familiar faces on face-naming performance and the occurrence of TOTs. To explore this research question, visual fluency was manipulated by setting of the clarity of presented faces by pixilating a photograph (three levels: high, medium, and low). Participants first performed a name recall test for the famous faces and then judged if they were in a TOT if they failed to recall the name. The recognition test for the names of the faces was performed after the TOT judgment had been made. Results showed that the recall rate and TOT experience rate were higher in the most visually fluent face condition than the other two conditions, and the recall rate was higher in the medium level of visual fluency condition than in the lowest condition. Participants reported more TOTs in the highest visual fluency condition than in the lowest one, and they experienced more TOTs in the medium visual fluency condition than in the lowest fluency condition as well. The recognition test results differed from recall and TOT results in that visual clarity did not affect face name recognition. In addition, regardless of clarity, the recognition rate for the stimuli for which the participants experienced TOTs was higher than that of the no-TOT stimuli. In summary, these results showed that the recall rate and TOT experience rate are higher for the faces that had higher clarity than those that did not. Thus, these findings support the main hypothesis of this study. This study confirmed that visually fluent processing significantly influenced TOT experiences.

That the fluency of the cue influences TOTs has been previously shown with paired-associate words (Metcalfe et al. 1993) and fictional animals (Schwartz and Smith 1997). Metcalfe et al. (1993) showed that the number of cue repetitions increased TOTs, irrespective of target learning. Schwartz and Smith (1997) found that cue priming increased the reported TOTs in the condition with minimal information about the targets, but cue priming did not significantly affect TOTs’ occurrence under the conditions in which more information was provided. However, there has been no research examining if fluency of processing faces affects TOTs for the names of those faces. In the current experiment, we showed that increasing the fluency of a face stimulus leads to more TOTs for the name. Moreover, unlike the earlier studies, this study shows an effect of cue fluency without varying the amount of time the cue is seen in the experimental context. We also think that there should be attention paid to cue factors in TOTs, as much of the evidence for retrieved information has been challenged (Huebert et al. 2022).

**Figure 8:**
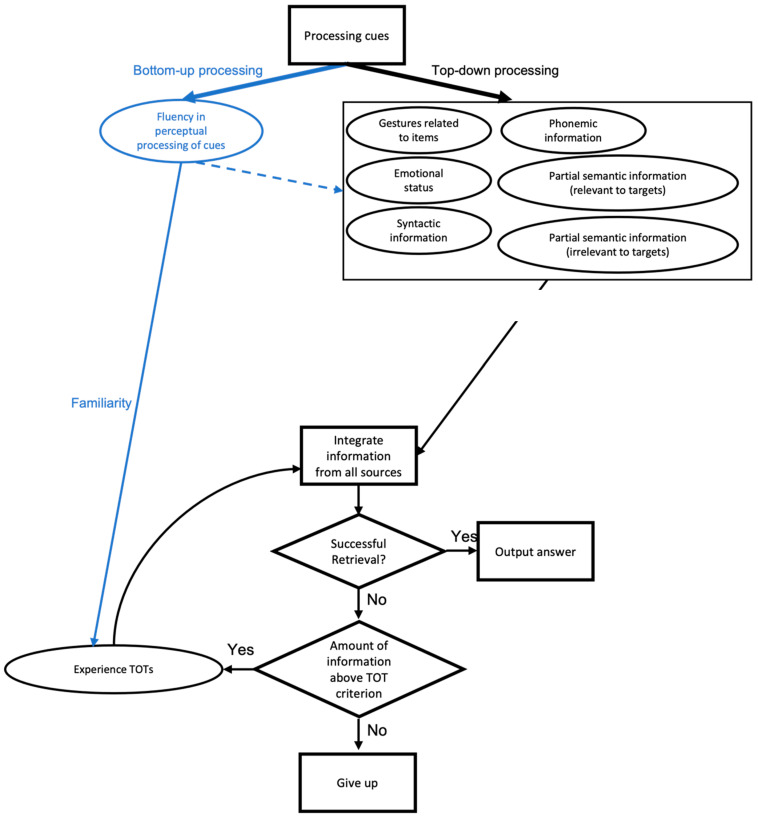
Modified retrieval modeling by the heuristic–metacognitive account (black: modeling that combines the models of Schwartz and Metcalfe (2011) and Schwartz and Pournaghdali (2021). Blue: new hypothesis presented here).

In the Schwartz and Metcalfe (2011) model, TOTs occur when a combination of heuristic cues add up to trigger a TOT. In the model, there are no weights given to different factors, nor does it distinguish between early factors and later factors causing TOTs. TOTs are simply determined by the sum of the TOT-inducing factors. In the current study, we show that the bottom-up processing of cue stimuli affects TOT experiences, as the clarity of the faces influences the rate of TOTs. It is likely that fluent perceptual processing leads to a subjective experience of familiarity for the cue (i.e., the face) that is attributed to memory for the name. Kleider and Goldinger (2004) stated that fluency in perceptual processing can be used as a memory cue when familiarity is a key determinant of recognition, and they found that the more perceptually fluently a face is processed, the more familiar it feels. Indeed, this study also showed higher perceived familiarity at the high-clarity level than at the low-clarity level. Although our data support the heuristic–metacognitive account for the cause of TOTs (Schwartz and Metcalfe 2011), we think it may be possible to decouple early (bottom-up) and late (top-down) causes of the TOT. The bottom-up processes derive from fluent processing of the cue, whereas top-down processes may include such factors as retrieval of related information or the retrieval when having previously been in a TOT for that item. Figure 8 illustrates this difference. In Figure 8, the right side represents the original model of Schwartz and Metcalfe (2011). On the left, however, is the view that TOTs may be caused early by cue-based factors and then influenced later by other factors, such as the retrieval of related information.

Turning now to recall, we found that clearer faces were better cues for recalling the name than were the faces that were less clear. However, although recall was also affected by face clarity, recognition was not. There were no differences in recognition performance across clarity conditions. For recall, participants relied on the visual image of the face to prompt recall. Apparently, clarity led to stronger associations between the face and a name and recall increased. However, for recognition, participants saw names, and it is likely that this served as a strong cue to retrieval, which over-rode clarity as a factor.

For the recognition test, we presented two alternatives for possible names in the recognition test. It is likely that the participants used the recognition heuristic for the two alternatives as discussed by Gigerenzer and Gaissmaier (2011). They argued that people likely infer that one alternative is more reasonable if one is recognized and the other is not. According to their argument, participants might choose one of the two alternative names that would be better recognized when matched to the corresponding face, and the role of the visual fluency of the cue would be small in this case. Likewise, Tulving and Thomson (1973) proposed that name alternatives may act as useful retrieval cues, which can facilitate recognition by increasing the probability of finding desired information while using their memory. To sum up, in the recall and TOT phases, visual fluency aided recall and served as a heuristic to determine a TOT state. However, in the recognition phase, participants likely relied more on the plausibility of name candidates rather than visual fluency of the face.

Another novel aspect of this study was asking participants to determine how they came to their recognition decision, that is, the selection justification task. We gave participants four options—(1) Now I remember the name, (2) I guessed the name and I do not know who the person is, (3) I guessed the name but I do know who the person is, and (4) I am sure the other name option is not correct. We found that there was an effect of being in a TOT. Participants reported to have recognized the famous names during TOTs, and they reported that they guessed the names when TOTs were absent. We found that, although it occurred in a small proportion of trials, participants reported more that they “guessed the names even though they knew who the person was” during TOTs than during n-TOTs. This is consistent with Cleary’s work (Cleary 2019; Cleary and Claxton 2015; Huebert et al. 2022) in which a TOT state activates a positive expectation about the target, leading people, in this case, to believe that they had guessed at the target name. Participants might have thought that they would be able to guess the names if they only recalled the faces’ first names or their characters’ names because this experiment asked for the last names of famous faces. For instance, when the participant looked at Benedict Cumberbatch’s face, if the person recalled Benedict but not Cumberbatch, then they might feel that they are more likely to have recognized the last name. Similarly, a TOT may arise based on the fluent processing for Benedict Cumberbatch, but the participant’s TOT may have been directed at the name “Dr. Strange,” one of the characters played by Cumberbatch, rather than the actor’s actual last name.

Given the result of this study, there are several promising ideas for future research. First, due to the experiment design in this study it is difficult to distinguish whether cue fluency triggered TOTs before other factors that cause TOTs (see the blue dashed line in Figure 8) or the cue fluency results in TOTs at the same time as other factors (see the blue solid line in Figure 8). One way to test for this can be presenting foil stimuli with different levels of clarity. For example, participants can be asked if they experience TOT after adding a condition that includes non-famous faces as well as famous faces to the experiment. If they are affected by fluent processing regardless of whether they knew the faces before or not, they would report more TOTs on clearer faces even for the non-famous faces. This result would demonstrate TOT experience can occur for bottom-up cue information even when there is no other source of information. Following the work of Benjamin (2005) and Koriat and Levy-Sadot (2001), cue factors work early and when time is pressed, but as people have more time to work on retrieval, later slower retrieval-based processes take effect. Thus, we could also force people to make rapid TOT decisions and see if these are more influenced by cue factors than a less rapid TOT choice. That is, different factors may influence TOTs at different times after unsuccessful recall. This has been shown for other metacognitive judgments. Undorf et al. (2018), for example, demonstrated that in the presence of multiple cues during the learning phase, people adaptively integrate these cues when making judgments about the chance to recall what they learned. Thus, it may be that people use different cues for inferring TOTs under different circumstances.

Taken together, the findings of this study demonstrate that fluent processing of familiar faces has an impact on name recall but not name recognition. It seems that people adaptively relied on available cues for retrieval. Participants utilized visual fluency as a retrieval strategy in the recall but used name alternatives as the strategy in the recognition phase. More fluent faces were better cues for the recall of the person’s name but not for recognition. Consistent with the original hypothesis, the visual fluency of the cue influenced TOTs for the target. Thus, this study presents data that are consistent with heuristic models of TOTs but extend the findings to the fluent processing of faces.

## Data Availability

The authors are working on making the data available.

## Note

1 We also conducted gamma correlation analyses to examine the relation between the number of TOTs to recognition of all unrecalled responses (see Nelson 1984). There were no differences in the gamma correlation across clarity conditions, F < 1. Each condition was significantly higher than zero, however, demonstrating the TOTs were accurate at predicting recognition performance. For the low clarity condition, the gamma correlation was 0.548 t(428) = 31.009. For the medium clarity condition, the gamma correlation was 0.565, t(428) = 32.664. For the high-clarity condition, the gamma correlation was 0.545, t(428) = 31.868.
